# Reverse Takotsubo Cardiomyopathy Following Exploratory Laparotomy

**DOI:** 10.1177/2324709618757259

**Published:** 2018-03-05

**Authors:** Tamer Akel, Emad Barsoum, Jad Mroue, Nikhil Nalluri, Frank Tamburino, Marc Bogin

**Affiliations:** 1 Staten Island University Hospital, Staten Island, NY, USA

**Keywords:** reverse takotsubo, cardiomyopathy, postoperative

## Abstract

Reverse takotsubo cardiomyopathy is an uncommon cardiomyopathy characterized by reversible regional wall motion abnormalities in the basilar segment of the left ventricle. This happens in the absence of any coronary artery pathology. Although it shares some pathogenic mechanisms with its more common variant, takotsubo cardiomyopathy, differences exist in terms of echocardiographic features, demographics, clinical manifestations, laboratory features, and prognosis. Cases of postoperative reverse takotsubo cardiomyopathy are less described in the literature. Herein, we report a case of reverse takotsubo cardiomyopathy in a 44-year-old woman occurring after exploratory laparotomy.

## Introduction

Takotsubo cardiomyopathy (TTC), also called “broken heart syndrome,” “transient apical ballooning,” and “stress cardiomyopathy,” is an acute cardiac syndrome that mimics myocardial infarction and characterized by transient cardiac wall motion abnormalities. This occurs in the absence of any coronary artery obstruction or acute plaque rupture.^1,2^ In most cases of TTC, the cardiac wall motion abnormality does not follow a single epicardial coronary artery territory. It is usually characterized by depressed function or akinesis of the mid and apical segments of the left ventricle along with hyperkinesis of the basal walls.^1^ On the other hand, reverse TTC (r-TTC), or inverted TTC, has been recognized as a variant with a hypocontractile ventricular basal segment along with a hypercontractile apex.^2^ Cases of r-TTC following surgery seems to be unusual and rare.^3,4^ In this article, we report a case of r-TTC in a patient who underwent exploratory laparotomy for small bowel obstruction.

## Case Presentation

A 44-year-old female patient with a known history of multiple sclerosis (MS) maintained on immunomodulatory agents presented to our emergency department with abdominal pain, nausea, and vomiting. Her past surgical history was only remarkable for appendectomy and partial small bowel resection. The patient was diagnosed with small bowel obstruction after a computed tomography (CT) scan of her abdomen was performed. She was admitted to the surgical service subsequently. However, her clinical status deteriorated and she underwent exploratory laparotomy on the next day. One day after surgery, she started experiencing shortness of breath. Her physical examination was otherwise unremarkable. An electrocardiogram was remarkable only for sinus tachycardia. A CT angiography of the chest for suspected pulmonary embolism was performed and was unremarkable. Serum troponin-T levels done were elevated and peaked at 2.19 ng/mL (reference range: <0.05 ng/mL). CT angiography of the coronary arteries was normal with a calcium score of zero. Echocardiographic examination done revealed a hyperkinetic apical wall along with a hypokinetic basilar wall of the myocardium suggestive of r-TTC with a left ventricular ejection fraction (LVeF) of 30% (Video 1; available in the online version of the article). Therapy with a β-blocker and an angiotensin-converting-enzyme inhibitor was initiated and the patient was discharged on postoperative day 8 with a persistent LVeF of 30%. A follow-up cardiac magnetic resonance imaging was done 14 days later after discharge revealed an improvement in the myocardial function with an LVeF of 54%. A repeat echocardiography also showed normalization of the LVeF.

## Discussion

The pathogenesis of both TTC and r-TTC is not well understood. Several mechanisms were postulated. The most widely accepted underlying etiological mechanism behind both types is sympathetic nervous system overactivation. Among the various neurochemical substances associated with cardiac wall motion abnormalities, epinephrine and norepinephrine seem to be the most crucial. This catecholamine surge is believed to mediate a vascular dysfunction leading to coronary artery vasospasm, microvascular dysfunction, hyperdynamic contractility, and direct myocardial toxicity via free radicals formation.^5^

Furthermore, there seems to be a role in protein signaling within the myocardial cells that mediates a paradoxical negative inotropic effect to protect against the intense activation of β-adrenoceptors. This effect is greatest at the apical myocardium where the β-adrenoceptor density is highest.^5^ This has been also proven by 123-meta-iodobenzylguanidine myocardial scintigraphy that implied more myocardial sympathetic innervation in the apex.^6^ This might explain the myocardial stunning affecting the apical wall in TTC. However, it does not explain the hyperkinetic apical wall motion in r-TTC, neither the hypokinesis of the basal wall. It has also been postulated that as catecholamine levels subside after a surge, a quicker apical recovery might happen leading to r-TTC pattern.^6^ Nevertheless, this again does not explain the hypokinesis observed in the basal wall.

Additionally, it is worth noting that certain clinical features differ between TTC and r-TTC.^2,7^ Song et al^8^ and Ramaraj et al^9^ observed that in r-TTC patients are usually younger, tend to have a lower LVeF, and sustains a quicker myocardial recovery in comparison to TTC. Moreover, since the basilar part of the ventricle is the main involved region in r-TTC, which has more myocardial tissue, cardiac biomarkers are usually more elevated in comparison to TTC.^2,9^

In the literature, most reported cases of r-TTC occurred after physical or emotional stress. Although surgery is considered a form of stress, it is less described. Nevertheless, it remains unclear whether r-TTC occurring after surgery is secondary to physical or emotional stress solely, or whether there is a role for anesthetic agents in triggering this cardiomyopathy. Furthermore, TTC and r-TTC have been reported in several neurological diseases such as subarachnoid hemorrhage, seizure, and ischemic stroke but rarely in MS patients.^10-13^ Biesbroek et al^11^ and Kozu et al^12^ both reported r-TTC in association with new MS lesion occurrence. They have speculated a possible role for demyelinating brain lesions interfering with sympathetic nervous system regulation. On the other hand, Peller et al^10^ reported r-TTC in a patient with stable MS, which is similar to our case; however, our patient had the complication after surgery and without any acute electrocardiogram changes, in comparison to the previous cases. It is unclear in our case whether MS had a role in triggering r-TTC or whether surgery itself was the sole stress triggering factor.

## Conclusion

Reverse takotsubo cardiomyopathy is a rare type of stress-induced cardiomyopathy that is described mainly following neurological insults. Cases of r-TTC following surgery are less described in the literature. We presented a case of r-TTC in a patient with stable MS who underwent exploratory laparotomy for small bowel obstruction.

## Supplementary Material

Supplementary material
